# Genome sequence of *Bacillus altitudinis* HH-03 isolated from dairy pipeline

**DOI:** 10.1128/mra.00094-25

**Published:** 2025-07-22

**Authors:** Yan-Hong Lu, Xi-Bei Fan, Ning Lv, Yang Liu, Zhan-Hui Guo, Rui-Long Xie, Zhe-Xue Quan

**Affiliations:** 1 School of Life Sciences, Fudan University 12478, Shanghai, China; 2 Key Laboratory of Milk and Dairy Products Detection and Monitoring Technology, State Administration for Market Regulation, Shanghai, China; 3 Inner Mongolia Yili Industrial Group Co. Ltd. 499033 , Hohhot, China; Loyola University Chicago, Chicago, Illinois, USA

**Keywords:** *Bacillus*, genome, dairy pipeline

## Abstract

In this study, *Bacillus altitudinis* HH-03 was isolated from a dairy pipeline, and its genomic information was obtained by performing sequencing. The genome size of strain HH-03 was 3.85 Mbp with a GC content of 41.2%. Based on the annotation procedure, 4,010 protein-coding genes were identified. The genomic information of strain HH-03 will be helpful for the development of clean-in-place processes.

## ANNOUNCEMENT

Dairy products are susceptible to contamination by *Bacillus* during processing and production since *Bacillus* can form endospores and biofilms (1), possesses high heat resistance and dispersal abilities, and can grow and multiply in milk pipelines (2). A significantly elevated incidence of *Bacillus cereus* contamination was reported in unprocessed milk samples (3), and *Bacillus altitudinis* contamination was detected in pre-pasteurized milk (4).

We isolated heat-tolerant bacteria from the washed water of the milk pipeline in Hubei Province, China, after boiling the sample for 10 min and growing the bacteria on nutrient agar with manganese at 37°C for 2 days. Most colonies were identified as *Bacillus altitudinis* based on nearly complete 16S rRNA gene sequences obtained by amplification and sequencing. The strain HH-03 was streaked onto nutrient agar with manganese solid medium and incubated at 37°C for 24 h. A distinct single colony was subsequently inoculated into liquid nutrient agar with manganese and cultured at 37°C for 24 h . Then, the 16S rRNA gene sequences were amplified and sequenced to verify culture purity. We selected strain HH-03 for further genome analysis. Genomic DNA from the pure culture of strain HH-03 was extracted using the MagAttract HMW DNA Kit (Qiagen, USA). The sequencing library was constructed using the Transposase Enzyme Linked Long-read Sequencing (TELL-Seq) WGS Library Prep Kit (Universal Sequencing Technology, USA) and the sequencing primers provided by the TELL-Seq Illumina Sequencing Primer Kit (Universal Sequencing Technology, USA), following the manufacturer’s instructions (5). The libraries were sequenced on an Illumina NovaSeq 6000, 150 bp paired-end platform to obtain raw sequencing data. Quality control and assembly of the raw sequencing data of strain HH-03 were conducted using the TELL-Read and TELL-Link processes provided by Universal Sequencing Technology (5).

The 632 M raw reads were assembled *de novo* using TELL-Read with parameters “-s T507 -g NONE” and TELL-Link with “-p 507.” Contig sequencing depths were quantified using BWA (v0.7.17) (6), with the assembled sequences showing a mean sequencing depth of 112.

The obtained contigs were subjected to BLAST analysis against the NCBI database to identify contaminated sequences. Following the removal of contigs exhibiting inadequate sequencing depth below 80 and contamination, the strain HH-03 yielded 12 contigs, with the largest one being 3.82 Mbp in length.

**Fig 1:**
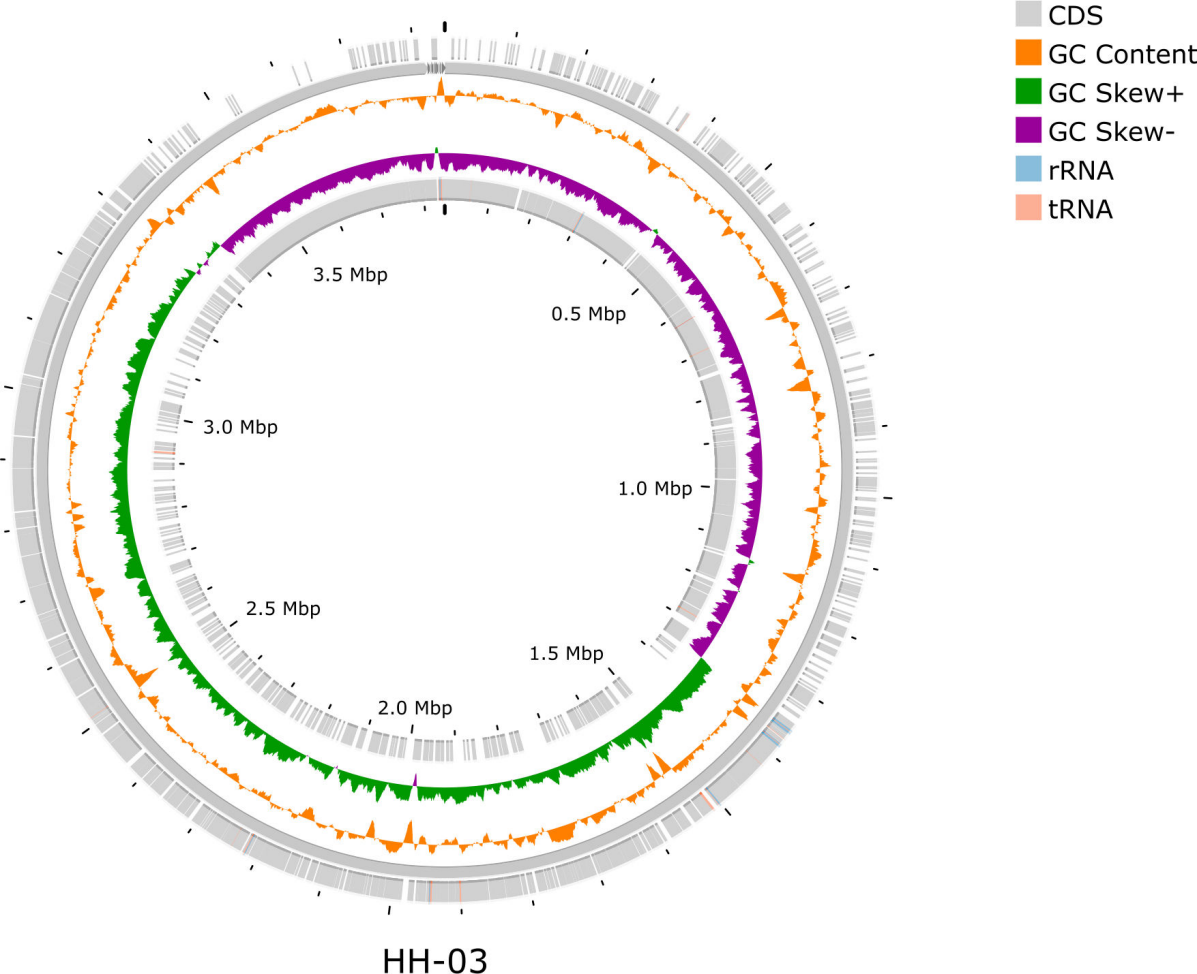
The figure represents the distribution of various genomic features, with the following color codes: gray: coding sequences (CDS); orange: GC content; green: positive GC skew (GC Skew+); purple: negative GC skew (GC Skew-); blue: rRNA genes; pink: tRNA genes.

To gain insight into the genome structure, the coding sequences of the positive and negative strands of the genome, the location of non-coding RNAs, and the spliced genome were plotted in a genome feature circle diagram. The spliced FASTA files were uploaded to CGViewer (7), an online genome feature map website, for online interactive operations, and the genomes were annotated using PGAP (v6.9) (8). The genomic circle diagram of strain HH-03 is shown in Fig. 1. The assembled genome size of the sample was 3,846,532 bp with a GC content of 41.2%. Additionally, the sample contained 3,796 coding sequences (with protein), 11 complete rRNA genes, and 75 tRNA genes.

Strain HH-03 was subjected to average nucleotide identity (ANI) analysis by using JSpeciesWS (9). The highest ANI value for strain HH-03 genome was 97.85% with *Bacillus altitudinis* (GCF_001191605.1), indicating that strain HH-03 should be identified as *Bacillus altitudinis*.

The genomic information of *Bacillus altitudinis* HH-03 will be helpful for the development of clean-in-place processes.

## Data Availability

*Bacillus altitudinis* HH-03 was deposited in Guangdong Microbial Culture Collection Center (GDMCC) under accession number GDMCC 1.5556. The genome sequence of strain HH-03 from the current study has been deposited in eLMSG (an eLibrary of Microbial Systematics and Genomics, https://www.biosino.org/elmsg/index) under accession number LMSG_G000053875.1 and in NCBI under accession number JBLADY000000000 (SRA accession number SRR32489392). Additionally, the assembled genome sequence is available in GenBank under accession number GCA_047204925.1.
